# Evaluation of the Anti-Inflammatory and Antioxidant Potential of *Cymbopogon citratus* Essential Oil in Zebrafish

**DOI:** 10.3390/ani14040581

**Published:** 2024-02-09

**Authors:** Kiara Cândido Duarte da Silva, William Franco Carneiro, Bárbara do Carmo Rodrigues Virote, Maria de Fátima Santos, João Paulo Lima de Oliveira, Tássia Flávia Dias Castro, Suzan Kelly Vilela Bertolucci, Luis David Solis Murgas

**Affiliations:** 1 Faculty of Animal Science and Veterinary Medicine (FZMV), Department of Veterinary Medicine, Federal University of Lavras, Lavras 37200-900, Minas Gerais, Brazil; kiaracandido@hotmail.com (K.C.D.d.S.); william.carneiro@ufla.br (W.F.C.); barbara-crv@hotmail.com (B.d.C.R.V.); 2 School of Agricultural Sciences of Lavras (ESAL), Department of Agriculture, Federal University of Lavras, Lavras 37200-900, Minas Gerais, Brazil; mariadefatimasmf@gmail.com (M.d.F.S.); joaopaulolimanut@gmail.com (J.P.L.d.O.); suzan@ufla.br (S.K.V.B.); 3 Institute of Biomedical Sciences II (ICBII), Universidade de São Paulo, São Paulo 05508-000, São Paulo, Brazil; tassia_fd@hotmail.com

**Keywords:** *Danio rerio*, free radicals, lemongrass, medicinal plant

## Abstract

**Simple Summary:**

Lemongrass (*Cymbopogon citratus*) is frequently consumed as an infusion because of its pharmacological properties, including suspected anti-inflammatory effects. In this study, we examined how lemongrass essential oil, a natural product, can help protect against inflammation and damage caused by stress in cells. We used zebrafish, a small fish often used in scientific research, to determine how different amounts of lemongrass oil affected their health. Our focus was on how the oil influenced the movement of certain immune cells (neutrophils), the healing of the fishes’ tail fin, and the health of their cells. We found that lemongrass oil, at all tested levels, reduced the movement of these immune cells. Interestingly, higher amounts of the oil slowed down the healing of the tail fin. We also noticed changes in the activities of some of the fishes’ protective enzymes, which are important for defending their cells against damage. Our study suggests that lemongrass oil could be useful for its anti-inflammatory properties and might help protect cells from certain types of stress. These findings are valuable because they can lead to a better understanding of natural remedies and their potential use in health and disease management.

**Abstract:**

This study explored the protective capacity of the essential oil (EO) of *Cymbopogon citratus* against oxidative stress induced by hydrogen peroxide (H_2_O_2_) and the inflammatory potential in zebrafish. Using five concentrations of EO (0.39, 0.78, 1.56, 3.12, and 6.25 μg/mL) in the presence of 7.5 mM H_2_O_2_, we analyzed the effects on neutrophil migration, caudal fin regeneration, cellular apoptosis, production of reactive oxygen species (ROS), and activities of the antioxidant enzymes superoxide dismutase (SOD), catalase (CAT), and glutathione S-transferase (GST) after 96 h of exposure. A significant decrease in neutrophil migration was observed in all EO treatments compared to the control. Higher concentrations of EO (3.12 and 6.25 μg/mL) resulted in a significant decrease in caudal fin regeneration compared to the control. SOD activity was reduced at all EO concentrations, CAT activity significantly decreased at 3.12 μg/mL, and GST activity increased at 0.78 μg/mL and 1.56 μg/mL, compared to the control group. No significant changes in ROS production were detected. A reduction in cellular apoptosis was evident at all EO concentrations, suggesting that *C*. *citratus* EO exhibits anti-inflammatory properties, influences regenerative processes, and protects against oxidative stress and apoptosis.

## 1. Introduction

The use of medicinal plants forms the cornerstone of traditional medicine, which has existed for millennia. Nonetheless, less than 10% of the current biodiversity has been explored for potential medicinal applications [1]. The World Health Organization (WHO) estimates that 80% of developing countries incorporate traditional medicine into their primary healthcare systems, with 85% using medicinal plants and plant extracts [2]. The appeal of medicinal plants can be attributed to several factors, such as their diversity, use as low-cost treatments, and accessibility [3]. Considering the potential advantages of these plants, it is important to reveal their therapeutic properties and expand our understanding of their role in contemporary medicine

*Cymbopogon citratus* (DC.) Stapf, commonly known as lemongrass, has a considerable impact on ethnopharmacological applications worldwide [4]. With its geographical distribution spanning Asia, Africa, and Australia, each region harnesses its therapeutic attributes within the framework of traditional medical practices. In these geographical contexts, *C. citratus* manifests in a myriad of medicinal roles. Typically, the applications of *C*. *citratus* involve the preparation of infusions and decoctions, leading to the development of therapeutics for managing febrile conditions and disorders of the gastrointestinal and central nervous systems [5]. Many of the medicinal properties of *C. citratus* arise from its essential oil (EO). This EO, derived from the plant’s secondary metabolic processes, has attracted scientific interest because of its potential as a promising source for novel pharmacological developments targeting conditions linked to oxidative stress and inflammation [4,6]. Given its wide-ranging therapeutic potential, a thorough scientific investigation of *C. citratus* EO (CEO) is essential.

Oxidative stress is caused by an imbalance between the production of reactive oxygen species (ROS) and the elimination of antioxidants. If not properly regulated, ROS can cause damage to proteins, lipids, and DNA, and induce cytokine production that increases inflammation, apoptosis, and necrosis [7]. Inflammation allows the immune system to remove harmful stimuli and initiate tissue repair. However, prolonged inflammation can also contribute to disease progression [8]. Oxidative stress and inflammation are closely intertwined—each can trigger and exacerbate the other [9]. Given the need for treatments that can disrupt this cycle, there is growing interest in plant derivatives that moderate oxidative stress and inflammation.

The multifaceted exploration of phytochemicals and compounds sourced from the natural milieu presents a challenge because of the polydispersity of metabolites, which could exhibit altered functionalities when removed from their inherent ecological niche [10]. The pursuit of identifying plant-based compounds with pharmaceutical potency necessitates a meticulous examination of various parameters including, but not limited to, cultivation practices, ethnopharmacological history, utility, processes of active compound isolation, and subsequent characterization. Moreover, integral components of this scrutinizing process include the appraisal of the drug’s potency and safety parameters, as well as undertaking both preclinical and clinical assessments [11]. The execution of rigorous toxicological assessments, employing animal models, is essential to identify the potential harmful consequences associated with these bioactive compounds. Furthermore, investigating the multiple chemical configurations in natural substances may lead to the discovery of novel molecules with pharmacological potential [12].

The zebrafish (*Danio rerio*) model offers many benefits for drug screening [13]. It serves as a viable alternative to traditional laboratory animals such as rats, mice, and rabbits [14] because of its morphological, genetic, and physiological parallels with humans [15]. The zebrafish model enables swift and accurate assessment of various substances and their potential impacts [16,17]. In addition, zebrafish are easy to handle, show rapid development, are cost-effective, and can be employed at all life stages [18]. In addition, zebrafish possess a sequenced genome [19], external reproduction, and transparent embryos [20]. Consequently, this model offers significant insights into the mechanisms of toxicity in medicinal plants and assists in the identification and discovery of new pharmaceuticals for the treatment of a range of diseases [14].

Due to the increasing demand for the identification of new bioactive compounds with therapeutic potential from natural sources, the present study aimed to evaluate the anti-inflammatory potential of *Cymbopogon citratus* essential oil (CEO) by analyzing neutrophil recruitment and caudal fin regeneration in zebrafish larvae. In addition, the protective effect of EO on oxidative stress, cell apoptosis, and the production of reactive oxygen species (ROS) induced by hydrogen peroxide is evaluated. Our core hypothesis posits that CEO could exhibit advantageous characteristics in modulating inflammatory responses and oxidative stress in zebrafish, potentially presenting promising therapeutic applications in human medicine.

## 2. Materials and Methods

### 2.1. Chemicals and Reagents

1-phenyl-2-thiourea (PTU), 1-Chloro-2,4-dinitrobenzene (CNDB), 2′,7′-dichlorofluorescein diacetate (DCF-DA), acridine orange, calcium chloride, nitroblue tetrazolium chloride (NBT), dimethyl sulfoxide (DMSO), dibasic potassium phosphate, monobasic potassium phosphate, riboflavin, and tricaine were purchased from Sigma (Darmstadt, Germany). Hydrogen peroxide (H_2_O_2_) and methionine were purchased from Synth. Ethylenediaminetetraacetic acid (EDTA) was purchased from Vetec (Duque de Caxias, Brazil). Ethyl alcohol, potassium chloride, sodium chloride, magnesium sulfate, and Sudan black were purchased from Êxodo Científica (Sumaré, Brazil). Bradford reagent was purchased from Perfyl Tech (São Bernardo do Campo, Brazil).

### 2.2. Plant Material and Obtaining Essential Oil

The *C. citratus* used in this study was sourced from the UFLA Medicinal Plants Garden, Lavras, MG, Brazil (latitude: 21°13′48.8″ S; longitude: 44°58′28.5″ W). A specimen was deposited in the UFLA Herbarium for reference (voucher specimen-ESAL18409). In this study, we followed the CEO extraction method detailed by Duarte da Silva [21]. Briefly, the process involved the steam distillation of 996.14 g of fresh leaves with eight liters of distilled water. The distillation was carried out over a period of three hours to ensure optimal extraction of the oil. Post distillation, the EO was purified using decantation. The extracted EO was then meticulously stored under controlled conditions until it was needed for further experimental use. In summary, the predominant constituents of the EO include geranial (43.72%) and neral (29%), which are isomers of citral, and myrcene (18.30%).

### 2.3. Zebrafish Maintenance and Embryo Collection

Adult wild-type zebrafish were raised and maintained in a recirculating water system specifically designed for the species (Hydrus ZEB-60, Alesco, SP, Brazil) as described by Duarte da Silva et al. [21]. Briefly, the culture conditions comprised a temperature of 28 ± 1 °C, a 14/10 h light/dark cycle, twice daily feeding with a flake diet, and daily provision of Artemia nauplii. All eggs were gathered from natural spawning events and kept in E3 medium (NaCl 5 mM, KCl 0.17 mM, CaCl_2_ 0.33 mM, MgSO_4_ 0.33 mM; pH 7.4). Harvested fertilized eggs were gently rinsed in E3 medium, screened under a microscope (CX31, Olympus, Tokyo, Japan), and selected for downstream assays under a 40× objective. Subsequently, 4 h post fertilization (hpf), embryos were singled out and transferred, in triplicates of 20 embryos per plate, into Petri dishes containing one of the CEO dilutions (0.39, 0.78, 1.56, 3.12, or 6.25 μg/mL) in 0.5% DMSO vehicle control. Culture plates were then incubated at 28.5 °C. Test concentrations were defined based on the basis of LC_50_ (9.02 µg/mL) data from our previous study [21].

### 2.4. Neutrophil Migration

To assess neutrophil migration, 20 embryos at 8 hpf were exposed to 0.003% 1-phenyl-2-thiourea (PTU; Sigma-Aldrich, St. Louis, MO, USA) to inhibit tyrosinase, a key enzyme in the melanogenic pathway. After 72 hpf, larvae were exposed to the determined concentrations of EO for 2 h before lesioning the caudal fin. In addition to evaluating neutrophil migration at each CEO concentration, we also assessed it in the control treatment and in a separate set of larvae exposed only to DMSO to discern the specific effects of CEO from those of the solvent. The larvae were then anesthetized with 0.016% tricaine, positioned in a Petri dish, and the caudal fin primordia were cut with a surgical scalpel blade just past the notochord with the aid of a stereomicroscope at 10× objective [22,23]. The larvae were returned to the tested solutions and incubated at 28 ± 1 °C for 6 h [24]. After 6 h, larvae were fixed overnight with 4% paraformaldehyde solution and subsequently stained with Sudan black for 20 min [24,25]. Larvae were washed in 70% ethanol to remove excess dye to facilitate the visualization of individual neutrophils [26]. Images were captured and counting was performed using a stereomicroscope at 10× objective (Olympus, model CX31).

### 2.5. Caudal Fin Regeneration

Twenty larvae from each treatment group, the control (unamputated and amputated only fish), DMSO, 0.39, 0.78, 1.56, 3.12, and 6.25 μg/mL groups, were used to analyze the caudal fin regeneration process, following the protocol of Sun et al. [27]. The embryos obtained were kept in E3 medium until 72 hpf. Larvae were anesthetized with 0.016% tricaine, added to a Petri dish, and the caudal fin primordia was cut with a surgical scalpel blade just after the notochord, under the aid of a stereomicroscope at 10× objective (Olympus, model CX31) [22,23]. Larvae were photographed immediately after amputation (0 h post amputation (hpa)) using a microscope at 10× objective (Olympus, CX3, Tokyo, Japan). The larvae were then placed in the respective OE concentrations in a 96-well plate (1 larva per well) with 200 μL and photographed again at 72 hpa. The regenerated area of the larval caudal fin was quantified using Motic Image Plus 3.0 software.

### 2.6. Antioxidant and Apoptotic Activity Induced by Hydrogen Peroxide (H_2_O_2_)

To evaluate the antioxidant and apoptotic effects of EO, hydrogen peroxide (H_2_O_2_) was used as an intracellular inducer of oxidative stress [28]. This compound readily diffuses across cell membranes, reacting with intracellular ions to generate hydroxyl radicals, which are highly reactive and induce cell death via oxidative signaling [29,30,31,32]. The embryos were exposed to CEO concentrations at 1 hpf, followed by the addition of 7.5 mM of H_2_O_2_ to each plate. To evaluate the antioxidant and apoptotic effects, they were exposed to H_2_O_2_ until 96 hpf and 72 hpf, respectively [31,33].

Antioxidant enzyme activity was assessed using a pool of 20 larvae per replicate, totaling 60 larvae per treatment group: control (exposed and unexposed to H_2_O_2_), DMSO, 0.39, 0.78, 1.56, and 3.12 μg/mL groups. Larvae were exposed to CEO immediately post fertilization and maintained up to 96 hpf. The larvae were placed in microtubes containing 400 μL of cold phosphate-buffered saline (PBS) and homogenized with a glass rod. Subsequently, the homogenates were centrifuged at 4000× *g* at 4 °C for 15 min, and the supernatants were collected and stored at −20 °C until further analysis [34]. Protein concentration in the homogenates was determined according to the method described by Bradford [35] at 595 nm. Catalase (CAT; EC 1.11.1.6) activity was determined using the method of Claiborne [36]. A mixture of 30 μL of homogenized larval supernatant, 135 μL of PBS, and 135 μL of H_2_O_2_ was used. The decrease in absorbance was measured using a spectrophotometer at 240 nm for 2 min. The results are expressed in μ/mol of hydrogen peroxide degraded per minute per mg of protein. Superoxide dismutase (SOD; EC 1.15.1.1) activity was determined according to Song et al. [37]. An enzymatic assay solution was prepared containing 100 μL of PBS, 2 μL of EDTA, 40 μL of methionine, 11 μL of ultrapure water, and 15 μL of NBT. The reading was performed at 560 nm and the results were expressed in units of SOD per mg of protein [37,38]. Glutathione-S-transferase (GST; EC 2.5.1.18) activity was measured using the method proposed by Habig and Jakoby [39]. A total of 15 μL of homogenate, 50 μL of reduced glutathione-GSH, and 180 μL of CDNB was used. Absorbance reading was performed at 340 nm for 3 min using a spectrophotometer. The results are reported as units per mg of protein [40].

For apoptotic cell identification, at 72 hpf 20 larvae treated with CEO were washed twice with E3 medium, followed by staining with a solution containing 5 μg/mL of acridine orange (AO) for 20 min in a dark, room-temperature environment [41,42], as recently detailed by Duarte da Silva et al. [21]. Apoptotic cells were also assessed in the control groups (exposed and unexposed to H_2_O_2_ and exposed to DMSO).

For the quantification of reactive oxygen species (ROS), 20 larvae per treatment were selected. The determination of ROS was performed using a fluorescence probe method. In this procedure, the animals were incubated with DCF-DA at a concentration of 10 μM. The incubation was conducted for 20 min at room temperature and in dark conditions, according to the methodology established by Driver et al. [43] and described in further detail by Duarte da Silva et al. [21].

### 2.7. Statistical Analysis

To assess data normality, the Shapiro–Wilk test was initially employed for the data analysis. Once normal distribution was confirmed, an Analysis of Variance (ANOVA) was performed. Should any significant differences be identified, they were further investigated using Tukey’s post hoc test, with a predetermined significance threshold of 5%. All statistical analyses were carried out using Minitab^®^ software, version 18 (Minitab LLC, State College, PA, USA).

## 3. Results

### 3.1. Neutrophil Migration

**Figure 1:**
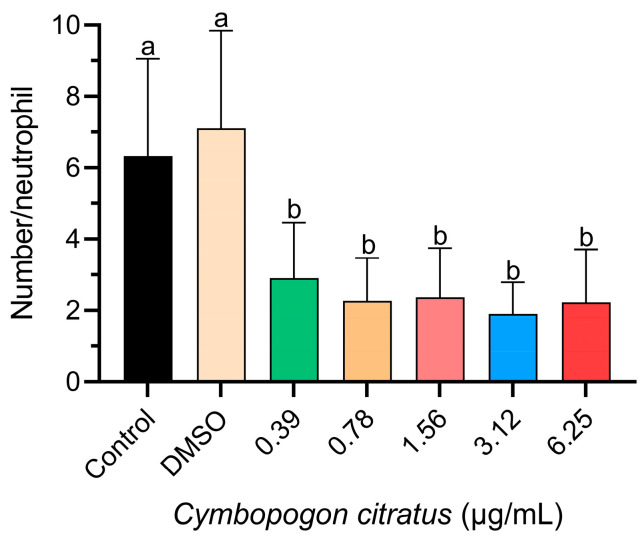
Influence of CEO on neutrophil migration. The data are represented as mean ± standard deviation (n = 20 larvae per treatment). Different superscript letters indicate significant differences between groups according to Tukey’s post hoc test.

In all treatment groups exposed to CEO, a significant decrease in neutrophil migration was observed compared to the control groups (Figure 1).

### 3.2. Tail Regeneration

**Figure 2:**
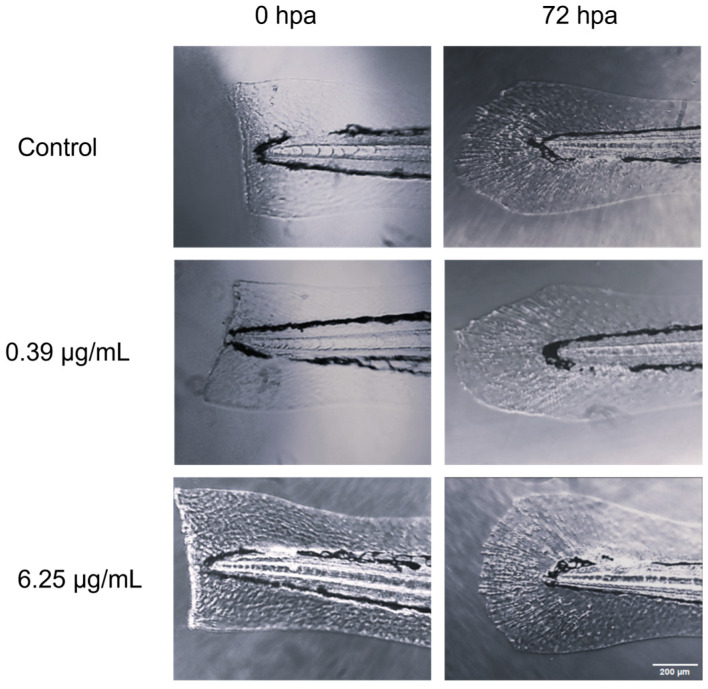
Comparative microscopic visualization of caudal fin regeneration in zebrafish larvae subjected to CEO treatment, captured at two time points: immediately post amputation (0 h) and 72 h post amputation.

**Figure 3:**
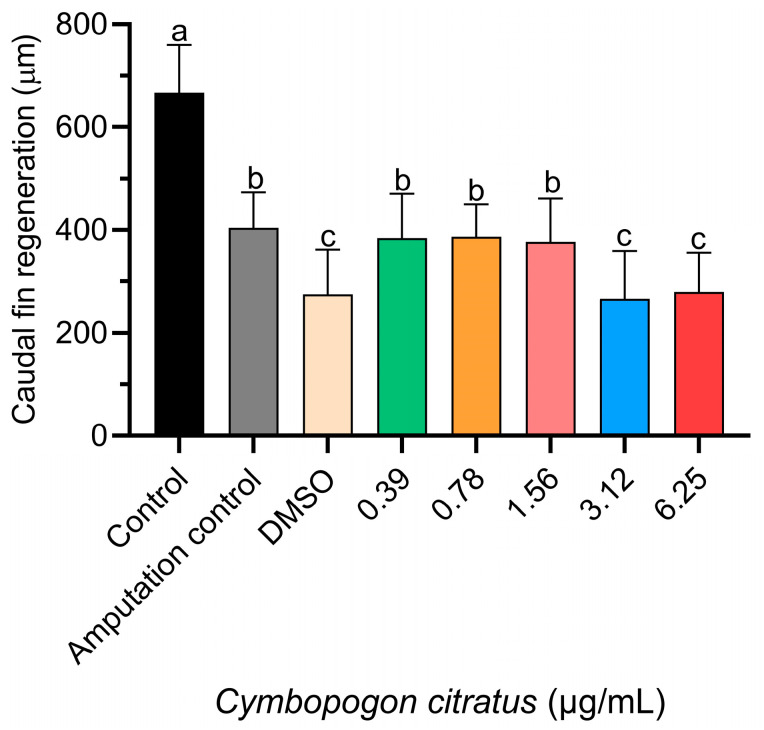
Influence of CEO on caudal fin regeneration in zebrafish larvae. Data presented as mean ± standard deviation (n = 20 larvae per treatment). Differing superscript letters denote significant statistical differences between treatment groups as determined by Tukey’s test.

Regarding tail fin regeneration (Figure 2), a significant decrease was observed in the groups treated with 3.12 and 6.25 µg/mL concentrations of EO compared to the other groups tested (Figure 3). However, the control group that had undergone caudal fin amputation showed a level of regeneration similar to that of the groups treated with 0.39, 0.78, and 1.56 µg/mL CEO, with no statistically significant differences observed among these groups (Figure 3).

### 3.3. Antioxidant Activity Induced by H_2_O_2_

**Figure 4:**
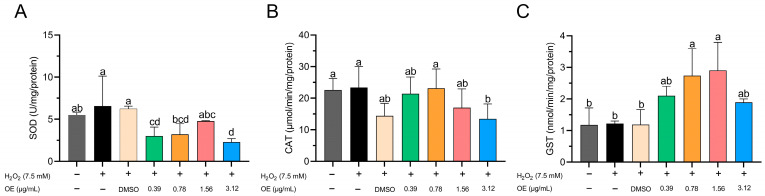
Influence of CEO on antioxidant enzyme activity: (**A**) superoxide dismutase (SOD), (**B**) catalase (CAT), and (**C**) glutathione s-transferase (GST) in zebrafish embryos/larvae at 96 h post fertilization (hpf). Data are depicted as mean ± standard deviation (n = 60 larvae per treatment). Differing superscript letters denote statistically significant differences among groups as per Tukey’s test.

SOD activity significantly decreased in groups treated with 0.39 and 3.12 µg/mL of CEO compared to the untreated control group, the group treated with H_2_O_2_, and the group treated with DMSO (Figure 4A). However, the groups treated with H_2_O_2_ and DMSO showed increased SOD activity compared to the EO-treated groups at concentrations of 0.39, 0.78, and 3.12 µg/mL (Figure 4A). The group treated with 3.12 µg/mL EO had significantly lower CAT activity compared to the untreated control group, the H_2_O_2_ control group, and the group treated with 0.78 µg/mL EO (Figure 4B). Conversely, GST activity tended to increase with higher EO concentrations to a concentration of 1.56 µg/mL. Notably, the highest GST activity was observed in the groups treated with 0.78 and 1.56 µg/mL EO, which surpassed the control groups (Figure 4C). In the context of the present study, it is pertinent to acknowledge that the evaluation of SOD, CAT, and GST activity at the uppermost concentration of 6.25 µg/mL was precluded due to the substantial mortality rates observed during the exposure phase to the essential oil (EO). This factor made it difficult to carry out a reliable assessment in this concentration range.

### 3.4. ROS and Apoptosis Induced by H_2_O_2_

**Figure 5:**
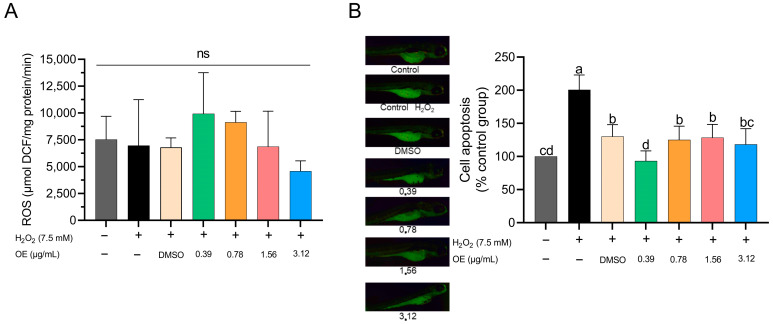
Effect of CEO on ROS production; no significance = NS (**A**). Cell apoptosis in zebrafish larvae subjected to H_2_O_2_ at 96 hpf (**B**). Data are expressed as mean ± standard deviation of the % of the control group (n = 60 larvae per treatment). Different superscript letters indicate significant differences between groups according to Tukey’s test.

The generation of ROS showed no significant differences between the tested CEO concentrations and the control groups (Figure 5A). In terms of apoptosis, the 0.39 µg/mL CEO concentration showed a decrease relative to other treatment groups; however, this change was not statistically significant compared to the control groups (Figure 5B). Interestingly, the 3.12 µg/mL CEO concentration also appeared to lower apoptosis levels, closely mirroring the results for the 0.78 and 1.56 µg/mL CEO concentrations (Figure 5B). It should be noted that for the highest concentration of 6.25 µg/mL, apoptosis evaluation was not feasible due to the elevated mortality rates observed during the experimental phase.

## 4. Discussion

Inflammation is the primary response of the innate immune system to the entry of pathogens, foreign bodies, or injuries into the tissue [44]. The primary goal of the inflammatory response is to localize and eliminate factors that interfere with homeostasis and initiate tissue restoration [45,46]. The inflammatory process involves tissue-resident cells, blood vessels, proteins, and immune cells [44,47]. During the recruitment process, macrophages and neutrophils are overactivated and induce the production of proinflammatory cytokines [48].

Neutrophil migration serves as the initial leukocyte response to tissue damage and infection and plays a crucial role in physiological responses to inflammation [49]. These cells function by localizing, phagocytosing, and eradicating micro-organisms by generating ROS and/or antibacterial proteins. The quantity of migrating leukocytes corresponds to the severity of inflammation [50]. However, unregulated and excessive neutrophil activity can result in persistent inflammation, tissue damage, and disease progression [51]. The present study demonstrates that all tested concentrations show potential anti-inflammatory effects by significantly decreasing neutrophil infiltration during inflammation compared with the control, underlining the therapeutic potential for controlling inflammatory processes. Leite et al. [52] demonstrated that the EO of *C. winterianus* inhibits carrageenan-induced neutrophil migration in mice in a dose-dependent manner. This suppression could be due to the inhibition of inflammatory mediators such as nitric oxide, prostaglandin E2, and cytokines like IL-1β, IL-6, and TNF-α that facilitate cell migration [53]. Certain molecules, specifically monoterpenes such as neral and geranial present in CEO, could contribute to the partial inhibition of inflammatory substances [54]. Citral has also been shown to limit the production of IL-1b, IL-6, and TNF-α [55,56]. Furthermore, EOs from lemongrass, geranium, and mint containing compounds such as citral, geranial, neral, and carvone have demonstrated inhibitory effects on proinflammatory cytokine production [57,58].

Neutrophil migration plays a critical role in tissue regeneration, a process through which damaged structures partially or completely regenerate. Zebrafish demonstrate a high capacity for epimorphic regeneration, including the ability to regenerate various complex structures such as the fins, heart, brain, and retina [59]. During regeneration, the wound epidermis and blastema are formed in response to tissue amputation. These structures, through coordinated actions, regulate cell proliferation and morphogenesis [22]. While appropriate neutrophil migration is important for mounting an immune response during regeneration, excess activity can contribute to tissue damage if improperly regulated [22]. In the present study, the lowest CEO concentrations did not significantly differ from the amputation control group; however, the highest concentrations (3.12 and 6.25 µg/mL) impeded the regeneration process. This inhibition may be related to the results of the embryotoxicity test, in which larvae showed morphological changes such as lordosis and deformity in the tail, as observed in another study [21], affecting the regeneration of the caudal fin, even with CEO showing a positive effect on neutrophil migration. Studies have shown that teratogenic effects can be attributed to the compounds present in CEO, such as citral, linalool, geraniol, geranyl acetate, and combinations of these compounds [60,61]. These compounds act rapidly in the organism and can affect respiration rates and cause damage to physiological processes and muscle activities, leading to permanent paralysis [62]. This evidence helps explain the inhibition of regeneration observed at higher concentrations, which could result from teratogenic effects disrupting physiological processes beyond the impact on productive neutrophil activity.

Therefore, signaling molecules known to regulate developmental processes have become the primary focus of regeneration research. One example is retinoic acid (RA), which is involved in several regenerative processes, such as the regeneration of fins and hearts in zebrafish [63]. Citral, one of the compounds in the CEO, has been investigated as an inhibitor of the RA signaling pathway, affecting tissue regeneration. Research on zebrafish larvae and adults exposed to citral has shown that it inhibits RA signaling, affecting wound epithelium, blastema formation, and fin regeneration [64]. Studies with the sea cucumber (*Holothuria glaberrima*) have demonstrated a significant reduction in intestinal rudiment, cell division, and differentiation in regenerated tissues compared with controls [65]. Furthermore, a study with axolotl (*Ambystoma mexicanum*) indicated that citral slowed the regeneration rate of forelimbs, severely affecting their pattern [66].

In the protochordate ascidian species (*Polyandrocarpa misakiensis*), citral inhibited the formation of the posterior half of the intestine (esophagus, stomach, and intestine) after amputation [67].

Living organisms undergo various chemical reactions, including electron oxidation and reduction, which are fundamental to metabolic processes [68,69]. These reactions can lead to the generation of free radicals, which are highly unstable and reactive molecules produced when electrons are uncoupled in the outer valence shell [68,70]. Oxidative stress results from either an excess production of these radicals or a deficiency in antioxidant systems [68,70]. Antioxidant enzymes, such as SOD, CAT, and GST, play vital roles in defending against oxidative stress induced by free radicals [71,72]. SOD converts superoxide (O_2_^−^) into hydrogen peroxide (H_2_O_2_), whereas CAT catalyzes the degradation of H_2_O_2_ into water and oxygen [73,74]. GST facilitates the biotransformation of exogenous and endogenous compounds, thereby detoxifying the body from ROS [75,76,77]. In our study, H_2_O_2_ acted as an intracellular promoter of oxidative stress, inducing cell apoptosis via oxidative signaling [29,32]. H_2_O_2_ is a stable molecule that diffuses rapidly across the cell membrane and is converted into highly reactive hydroxyl radicals [30]. In our study, the animals were pre-treated with CEO, resulting in a significant decrease in SOD levels. This suggests the antioxidant role of the EO and its potential to reduce the demand for SOD production in combating ROS. These observed decreases can be attributed to a mechanism of simultaneous antioxidant action between the compounds [78], such as monoterpenes, especially citral, which has shown antioxidant activity in previous studies [79,80]. A study involving *C. martinii* EO in rats demonstrated a reduction in SOD activity, which was attributed to the combined action of linalool and β-caryophyllene [78]. These compounds are also present in CEO, as described in our previous study [21]. CAT levels showed an increase, except for at the highest concentration, which displayed a significant decrease compared with the control. The CAT results can be explained by the dosages used. Concentrations lower than 3.12 μg/mL were not sufficient to inhibit the action of ROS present in the organism [78], since the induction of stress in the organism made too much H_2_O_2_ available in the medium. Terpenoids, the main components of EOs, are responsible for their antioxidant action [81,82], which may explain the decrease in SOD and CAT activity. Conversely, we observed a significant increase in GST levels, particularly at concentrations of 0.78 and 1.56 μg/mL. Buch et al. [83] evaluated the neuroprotective effect of *C. martinii* EO against global cerebral ischemia/reperfusion (I/R)-induced oxidative stress in rats and observed similar results for GST activity. These findings suggest that CEO may modify the activities of antioxidant enzymes, potentially acting as a protective agent against oxidative stress.

ROS overproduction is associated with cell death because ion imbalance can cause both direct and indirect damage to nucleic acids and change the structure and function of cellular lipids and proteins, ultimately leading to cell death [68,70]. In this study, we observed that none of the EO concentrations showed significant differences compared with the control groups. However, when examining apoptosis, all concentrations of CEO appeared to reduce apoptosis compared with the H_2_O_2_ control group. Despite no significant differences being observed in comparison with the control, the concentrations of 0.39 and 3.12 µg/mL appeared to decrease cell apoptosis. Moreover, the 3.12 µg/mL concentration showed effects similar to those of the 0.78 and 1.56 µg/mL concentrations and DMSO. The composition of CEO may help to inhibit cell apoptosis. This view is corroborated by studies that have demonstrated the beneficial effects of EOs on neuroprotection against glutamate-induced cell necrosis [84]. Furthermore, a study conducted with *Litsea cubeba*—which, like CEO, has citral as its primary active compound—showcased its renoprotective potential against renal inflammation due to its antiapoptotic effect [85]. Consequently, the CEO may have exerted a protective effect on H_2_O_2_-exposed larvae.

## 5. Conclusions

The results observed in our study suggest that CEO has potential anti-inflammatory properties and can act protectively against free radicals. These findings support the relatively scarce literature on the safe use of CEO. Given these encouraging results, we strongly recommend further exploration of CEO’s potential effects in animal models for a comprehensive understanding of its therapeutic implications.

## Data Availability

Data are contained within the article.
